# An analysis of segmentation dynamics throughout embryogenesis in the centipede *Strigamia maritima*

**DOI:** 10.1186/1741-7007-11-112

**Published:** 2013-11-29

**Authors:** Carlo Brena, Michael Akam

**Affiliations:** 1 Laboratory for Development and Evolution, Department of Zoology, University of Cambridge, Downing Street, Cambridge CB2 3EJ, UK

**Keywords:** Segmentation clock, Oscillation, Pair-rule genes, Single segment patterning, Arthropod, Evolution

## Abstract

**Background:**

Most segmented animals add segments sequentially as the animal grows. In vertebrates, segment patterning depends on oscillations of gene expression coordinated as travelling waves in the posterior, unsegmented mesoderm. Recently, waves of segmentation gene expression have been clearly documented in insects. However, it remains unclear whether cyclic gene activity is widespread across arthropods, and possibly ancestral among segmented animals. Previous studies have suggested that a segmentation oscillator may exist in *Strigamia*, an arthropod only distantly related to insects, but further evidence is needed to document this.

**Results:**

Using the genes *even skipped* and *Delta* as representative of genes involved in segment patterning in insects and in vertebrates, respectively, we have carried out a detailed analysis of the spatio-temporal dynamics of gene expression throughout the process of segment patterning in *Strigamia*. We show that a segmentation clock is involved in segment formation: most segments are generated by cycles of dynamic gene activity that generate a pattern of double segment periodicity, which is only later resolved to the definitive single segment pattern. However, not all segments are generated by this process. The most posterior segments are added individually from a localized sub-terminal area of the embryo, without prior pair-rule patterning.

**Conclusions:**

Our data suggest that dynamic patterning of gene expression may be widespread among the arthropods, but that a single network of segmentation genes can generate either oscillatory behavior at pair-rule periodicity or direct single segment patterning, at different stages of embryogenesis.

## Background

Most arthropods, like most other segmented animals, couple the processes of segmentation and growth, adding segments progressively to the posterior of the body. This process is termed short germ or sequential segmentation, to distinguish it from the long germ mode of segmentation seen in *Drosophila,* where all segments are generated simultaneously by a cascade of genetic interactions taking place in the blastoderm [1].

Despite considerable effort in recent years, we still understand little of the mechanisms that drive segmentation in sequentially segmenting arthropods. Particular interest has focused on the nature of the so-called “growth zone”, or “segment addition zone”, a region of apparently undifferentiated tissue at the posterior of the embryo from which segments emerge. In all of the short germ arthropods so far examined, homologues of the *Drosophila* pair-rule segmentation genes are expressed in this region, often in rather broad domains around the site of invagination of the proctodeum [2]. Patterned stripes of gene expression emerge from this posterior domain, through the apparent repression of expression in the interstripe regions. One major concern of this paper is the nature of the patterning process in this posterior region, and in particular, whether dynamic oscillations of gene expression precede segment patterning. Such cyclic gene expression has recently been documented unambiguously in one insect [3,4], suggesting parallels between segment patterning in short germ insects, and somite patterning in vertebrates [5].

In some arthropods (for example, the myriapods *Lithobius* and *Glomeris*, the spider *Cupiennius*), this initial pattern appears to have a single segment periodicity, as defined by the subsequent expression of *engrailed* and other segment polarity genes in register with these primary stripes, and by the appearance of definitive segment morphology shortly afterwards [6-8]. In other cases, including several well documented cases in short germ insects (*Tribolium*, *Schistocerca*), the earliest stripes to resolve are at a double segment or “pair-rule” periodicity, just as they are in *Drosophila*[9,10]. The single segment repeat is then generated either by the subsequent splitting of these stripes, or by the intercalation of secondary stripes between them, before the appearance of definitive segment pattern defined by *engrailed* and segment morphology.

The geophilomorph centipede *Strigamia maritima* provides a particularly clear example of double segment patterning [11,12]. In most short germ arthropods the growth zone contains relatively few cells, and pair-rule stripes resolve to a single segment shortly after they have appeared. In *Strigamia*, a large population of unpatterned cells is generated before segmentation starts, forming a large posterior disc. Within this population, several cycles of the “pair-rule” pattern persist before resolution to the single segment pattern, particularly during the early stages of trunk segment addition. We have previously documented this pattern for homologues of the pair-rule genes, including *even-skipped*, *hairy*, *runt* and *oddskipped*, and also for genes of the Notch signaling pathway - most strikingly, the gene encoding the Notch ligand *Delta*[12,13].

In all of these cases, the early “pair-rule” patterning of the genes extends as complete rings around a focal point: initially this focus is marked by an area of reduced cell density that we interpret as being the blastopore, based on morphology [14] and on gene expression data (Jack Green, unpublished data). Later, the proctodeum will invaginate close to this point, and the pattern becomes centered on the proctodeum. Within the area closest to the center of this pattern, gene expression appears highly variable, even in embryos of similar age. We have proposed [11,12] that gene expression may be dynamic in this “peri-proctodeal” area, with cells near the proctodeum showing oscillating gene activity, and a pattern emerging through the fixation of a travelling wave of gene expression to give rise to a static pattern of cell states in the transition zone, where the germ band emerges, before a further round of gene interactions defines the single segment pattern.

However, interpretation of the observed gene expression patterns is complicated by the accompanying movement of cells, as the posterior disc converges to form the germ band, and as anterior parts of the germ band condense towards the head [14]. It has not previously been clear to what extent patterns of transcript accumulation in the posterior disc and emerging germ band reflect dynamic aspects of gene expression, and to what extent they reflect these cell movements.

In this paper we document more robustly the evidence for dynamic patterns, using embryos that are staged by independent criteria to different phases of a single cycle of gene activity. We define more precisely the limits of dynamic gene expression, concluding that this is limited to the region within, and including, the first resolved “pair-rule” stripe, but that the movement of the remainder of the pattern is largely the result of cell movement.

We extend our observations to earlier stages of patterning, when the head segments are being defined. Using the *even-skipped* genes as representative of the pair-rule gene network, and the *Delta* gene as representative of the Notch signaling pathway, we show that both of these pathways are apparently involved in the patterning of all segments from the intercalary back, and that the earliest aspects of segment patterning appear to involve dynamic gene activity qualitatively similar to the patterning of the trunk.

We also show that the relative timing of double and single segment patterning shifts as more trunk segments are added, until at about the time that the 39^th^ segment is patterned, the oscillation that generates double segment periodicity appears to shut off; the last 10 or so segments resolve singly from a domain of ubiquitous and continuous posterior *even-skipped* expression.

## Results

Segmentation in *Strigamia* proceeds from anterior to posterior [14-16]. Five segments of the head appear first during stage 3 and then, after a short pause, leg-bearing segments (LBS) appear in sequence, initially at a uniform rate of 1 segment every 3.2 hours until about 39 LBS are visible at the end of stage 4. Thereafter, segments are added much more slowly, with the process pausing completely during the movements of germ band flexure in stage 6. The final leg-bearing segment is not demarcated until shortly before hatching.

In this paper, we have used the genes *Delta*, *even-skipped* (*eve1* and *eve2*) and *engrailed* to monitor the progress of this molecular patterning. *Delta* is a marker for cell interaction processes that are known to be important in the co-ordination of vertebrate segmentation, as well as in many other developmental processes; *eve* is a member of the primary pair-rule gene set that generates the first periodic, double segment pattern in *Drosophila*. It is also expressed later during *Drosophila* segmentation, with single segment periodicity. *Engrailed* is a widely used marker for the definitive segment pattern. It is expressed in the posterior part of each segment throughout the arthropods.

**Figure 1:**
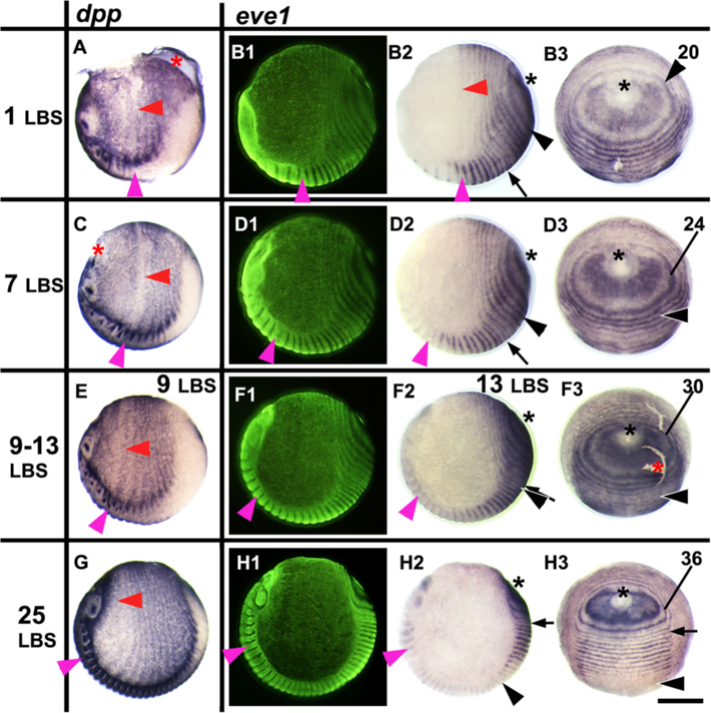
***eve1 *expression in relation to the movement of tissues during stages 3 to 4.2.** Embryos are aligned from top to bottom according to developmental age, indicated by the number of the last formed leg-bearing segments (LBS). The left column shows embryos stained for *dpp*, in lateral view, head on the left, ventral side at the bottom **(A, C, E, G)**. The three columns on the right show the expression of *eve1***(B, D, F, H)**; on each row the same embryo is viewed (1) laterally with florescent nuclear staining (SYBR green), (2) in normal light from the same view, and (3) from a posterior orthogonal view. Primary rings of *eve1* expression surround the proctodeum (black asterisk). Numbers marking the primary rings indicate the LBS number to which each ring will eventually give rise. Primary rings (and intercalated *eve1* stripes) are compressed and bent by the extension of the germ band around the egg and by the reduction of the terminal disc. The forward expansion of the rings is associated with a contraction of the whole egg epithelium towards the cephalic region of the germ band. This is shown by the movement of the *dpp* stripes which persist at double segment periodicity throughout the extra-embryonic region and move with associated morphological segments. Magenta arrowhead marks the second LBS; red arrowhead marks the associated ring of *dpp***(A, C, E, G)** or *eve1* (B2). The last resolved *eve* ring in embryo B is that corresponding to the 20^th^ LBS (numbered black arrowhead). The location of this ring/stripe in embryos **D, F** and **H** is marked with a black arrowhead. The most recently resolved *eve* ring in these embryos is numbered. Black arrow: first appearance of intercalating single segment periodicity *eve1* stripe; red asterisk: broken epithelium . Scale bar: 400 μm.

**Figure 2:**
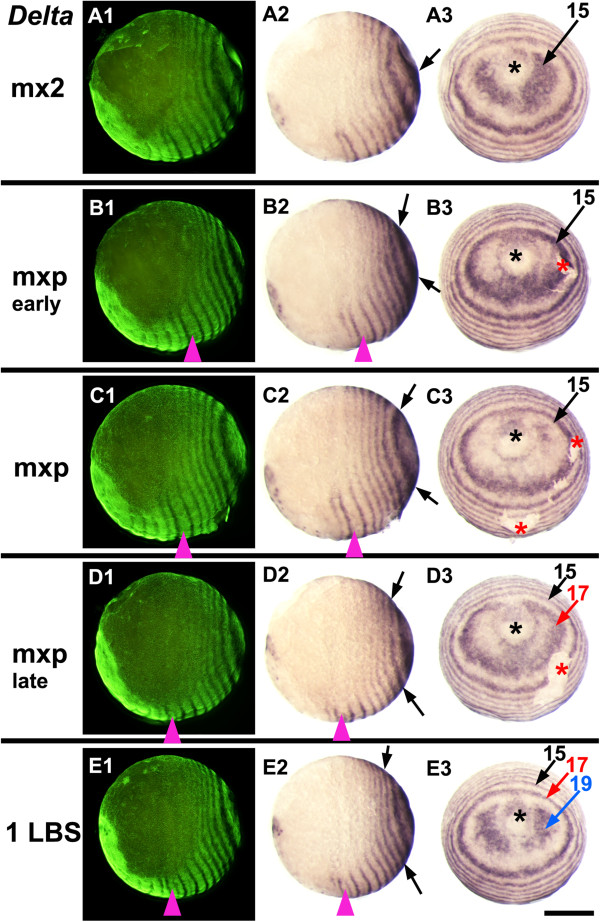
**Dynamics of *Delta *across two cycles of expression during stage 3.** Embryos are aligned from top to bottom according to developmental age, indicated on the left by the last formed morphological segment visible in each embryo (mx2: second maxilla, mxp: maxilliped, 1LBS: first leg-bearing segment). On each row the same embryo is viewed laterally with florescent nuclear staining (SYBR green) to show the morphology **(A1, B1, C1, D1, E1)**, in normal light from the same view **(A2, B2, C2, D2, E2)**, and from a posterior orthogonal view **(A3, B3, C3, D3, E3)**. Within this period, *Delta* expression around the proctodeum is dynamic, showing an oscillation of expression in each region of the peri-proctodeal area. Arrows of the same color mark a single cycle of expression, from its initiation as bilateral patches around the proctodeum (black asterisk) to its resolution as a primary pair-rule stripe. Magenta arrowhead marks the second LBS in lateral views: red asterisk marks artefactual breaks in the epithelium. Scale bar: 400 μm.

In *Strigamia*, both *Delta* and *eve* are expressed with a primary double segment periodicity as concentric rings around the site of invagination of the proctodeum, but out of phase with one another [12]. As the segmentation process continues, the appearance of intercalary stripes of *eve1* and *Delta* within the forming germ band defines the single segment repeat (Figures 1 and 2), and shortly thereafter, *engrailed* is activated in every segment [15]. A second *even-skipped* gene, *eve2*, is co-expressed with *eve1* during the primary, double segment phase, but is never activated in segmental stripes [13]. It ceases to be expressed as segments mature, except that it is transiently expressed specifically as a stripe in the antennal segment (see below).

### The dynamics of segmentation gene expression

These patterns of gene expression that presage segment formation arise near the posterior of the embryo, and then progressively move anteriorly as further segments are added behind them. This is visible, for example, in the series of embryos presented in Figure 2, where the primary *Delta* expression corresponding to LBS 15 arises as a patch adjacent to the proctodeum, becomes the first ring in slightly older embryos, and is then displaced anteriorly as the next ring resolves behind it.

**Figure 3:**
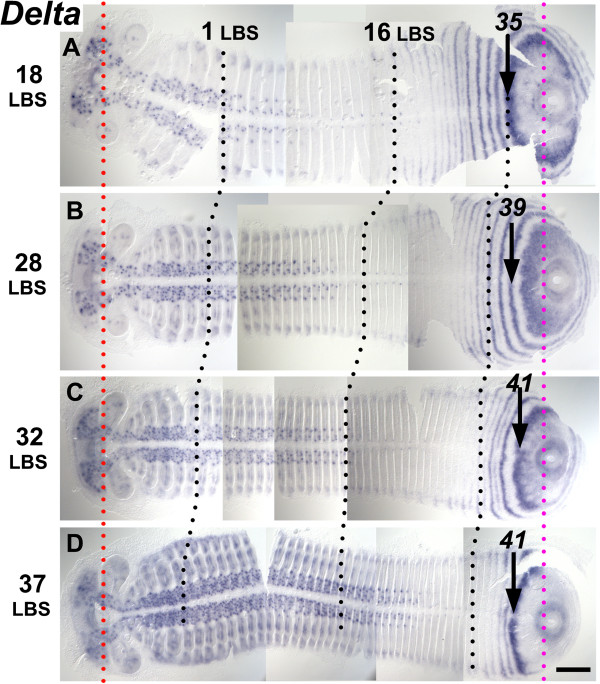
**Segmentation dynamics of *Delta *across mid-late stage 4.** Flat mounted embryos are aligned from top to bottom according to developmental age **(A, B, C, D)**, indicated on the left by the number of the last formed LBS. During this interval the distance from the stomodeum (red dotted line) to the proctodeum (magenta dotted line) is almost constant. The appearance of new stripes/rings of *Delta* expression and their forward movement is associated with the convergence of the germ band toward the anterior/cephalic region, as shown by black dotted lines marking the 1^st^ and the 16^th^ leg-bearing segment (LBS). Note, though, that the length of the germ band from the 16^th^ to the 35^th^ LBS (or the primary stripe that precedes it) is, if anything, expanding during this interval. The punctate expression of *Delta* in medial regions of anterior segments marks neural precursors arising in each segment. Scale bar: 200 μm.

In part, this movement results from a condensation of the whole surface epithelium of the egg towards the anterior. This is most obvious in the germ band, as the head extends forward and the first formed segments become relatively smaller (Figure 3; see also [14]). However, this movement also affects the territory outside the germ band. This is clear from the expression of a number of markers, including *decapentaplegic* (*dpp*) (Figure 1), which is expressed in a double segment pattern that mirrors earlier segmentation markers. This expression persists in the extra-embryonic territory after the resolution of segments in the germ band. The whole pattern can be seen to extend anteriorly in continuity with the morphological segments of the germ band.

We believe that this tissue movement may account for most of the movement of the gene expression patterns after the resolution of the first or second well-resolved rings around the proctodeum. It is presumably driven by cell proliferation in the posterior of the embryo, with space for the movement being generated by the continuing condensation of the head and anterior germ band, which continues throughout the period of segment addition.

**Figure 4:**
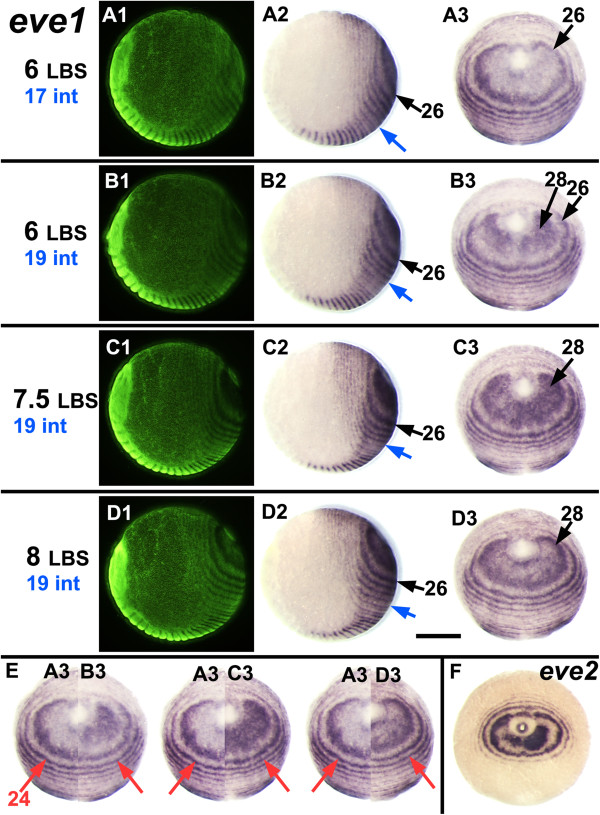
**A single cycle of *eve1 *expression. A-D:** Embryos very close in developmental age have been ordered from top to bottom according to the number of visible LBS (as indicated in black on the left) and the segmental position of the most recently intercalated *eve1* stripe (as indicated in blue on the left, and marked on the embryos with blue arrows). Together these markers provide an independent estimate of the developmental sequence for these four embryos. The series shows the oscillation of *eve1* expression in the periproctodeal region that generates the initial double periodicity stripe of *eve1* corresponding to the 28^th^ LBS, a period that corresponds to the formation of two segments. Each row presents three views of the same embryo as in Figure 2. **F:** Montages showing halves of embryos **B3**, **C3** and **D3** abutted with embryo **A3** to show how expression across the peri-proctodeal area expands while the external ring corresponding to the 24^th^ LBS (marked by red arrows) shows no detectable movement over this time window. **E:** Posterior view of an embryo showing bilateral asymmetry in the stage of the oscillation for *eve2*, resembling the juxtaposition of **A3** and **B3**. Scale bar: 400 μm. All embryos with ventral sides at the bottom.

However, this condensation of the germ band and surrounding epithelium is clearly not sufficient to account for the very dynamic patterns of gene expression seen in the region immediately around the site of invagination of the proctodeum (Figures 1, 2, 3, 4). We here define this region as the “peri-proctodeal region” to distinguish it from the larger area of the posterior disc, or growth zone, which encompasses the whole area of unsegmented tissue posterior to the emergence of segments. We have previously suggested that the appearance of the double segment pre-pattern in this peri-proctodeal region reflects a dynamic process of gene expression akin to that observed in the vertebrate pre-somitic mesoderm [11,12].

To demonstrate the generation of this pattern more precisely, Figure 4 illustrates four closely staged embryos from the same clutch that have been put into a developmental series by the number of leg-bearing segments (LBS) morphologically visible, and by the number of intercalated *eve1* stripes (panels A-D). Embryo A (six LBS, intercalated stripe 17 visible), has a relatively well resolved primary ring of *eve1* expression corresponding to LBS 26, but little or no *eve1* expression within this ring, closer to the proctodeum. The slightly older embryo B (also six LBS, but with intercalated stripe 19 visible) shows *eve1* expression in two bilateral patches adjacent to the proctodeum. This is the beginning of primary *eve1* ring 28. In embryo C, (with seven and possibly an eighth LBS visible), *eve1* expression now fills most of the area within ring 26, and in embryo D (eight LBS clearly visible, but still intercalated *eve1* stripe 19), the central region closest to the proctodeum is now clearing of *eve1* expression, defining the beginning of primary *eve1* stripe 28.

In Figure 4, panel E, embryo A has been montaged with bisected images of embryos B, C and D. These composite images suggest that primary *eve1* ring 26 resolves by the loss of expression from its internal margin, closer to the proctodeum, and by a limited expansion of the outer part of the ring. This expansion will presumably continue as subsequent primary rings appear, but any later expansion must be much slower than the resolution of the primary rings: on the timescale of this series, (and accepting the limitations of these composite embryo montages) there is no perceptible shift in the position of the older rings (for example, ring 24 marked by arrows; see also in Additional file 1: Movie 1).

At a low frequency in our population of eggs, we observe embryos that resemble these photographic montages. Embryo F in Figure 4, for example, is a slightly abnormal embryo, in which the development of the *eve* pattern (*eve2* in this case) is slightly asymmetric on the two sides. We suggest that this represents an embryo in which the right side is slightly ahead of the left in the cycle of segment generation, naturally reproducing the situation seen in the first montage.

**Figure 5:**
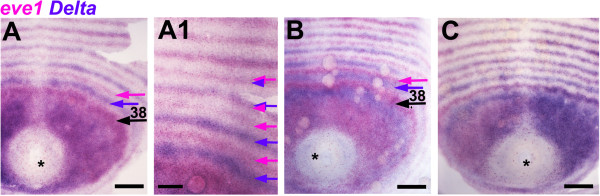
**Oscillation within posterior area of *Delta *and *eve.*** Posterior area, proctodeum included (asterisk), of flat mounted germ bands double stained for *eve1* and *Delta*, anterior to the top. Embryos **A** and **B**, very close in developmental age (around 24 LBS), show different phases of oscillation of *eve1* and *Delta* internal to the 38^th^ LBS ring of *eve1* expression (arrow). *Delta* is broadly expressed close to the proctodeum in **A**, largely overlapping with *eve*, but is down-regulated in this region in **B**, and resolving to a 39^th^ stripe. Once fully resolved, primary stripes of *eve* (magenta arrow) and *Delta* (blue arrow) are out of phase, while the following intercalated stripes are directly superposed (**A1**, high magnification of **A**). **C:** An abnormal asymmetric embryo showing different phases of oscillation on the two sides of the embryo. Asterisk marks the proctodeum. Scale bar: 100 μm in **A**, **B**, **C**; 50 μm in A1.

The appearance of primary rings of *Delta* shows this same pattern of cyclic activation initiating from bilateral patches (Figure 2). For *eve* and *Delta*, the fully resolved primary rings are out of phase (Figure 5); the expanding patterns close to the proctodeum do overlap transiently, but these too show different phases of oscillation, as illustrated in Figure 5 by two embryos about a half cycle apart (A, B) and by a bilaterally asymmetric embryo that shows a similar discrepancy between left and right sides (Figure 5C). Intercalated stripes, when they appear, are directly superposed - *eve* intercalated stripes within primary *Delta* rings, and vice-versa (Figure 5A1).

**Figure 6:**
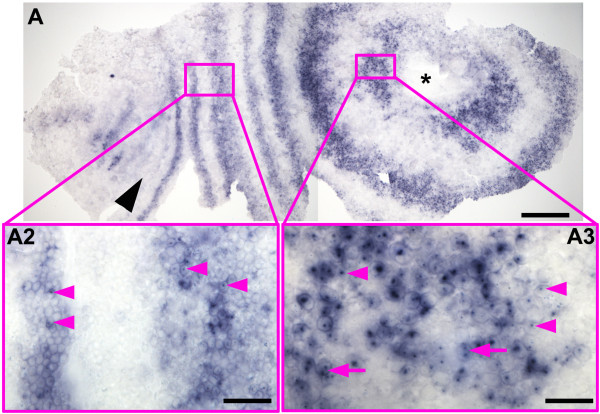
**Distribution of *Delta *nascent transcripts in an early stage 3 embryo.** An embryo at the onset of morphological segmentation hybridized with a *Delta* probe containing mostly intron sequences. This probe detects unprocessed nascent transcripts much more strongly than spliced cytoplasmic transcripts. At this early stage there are prominent nuclear dots of nascent transcript in many cells of the peri-proctodeal region (insert **A3**); double nuclear dots are often visible (magenta arrows), showing transcription associated with both copies of the gene. In more mature *Delta* stripes, transcript is still present at high levels in the cytoplasm, but nuclear dots are less frequent and less prominent (insert **A2**). (This embryo is slightly younger than that shown in Figure 2A. Only the mandibular segment was morphologically distinct (position marked by black arrowhead). Note that embryos at this stage extend around much of the egg, and are, therefore, distorted and split at the lateral margins when flattened. A forming head is on the left. Asterisk marks the proctodeum. Part of the head is missing. Scale bar in A: 200 μm, in **B:** 50 μm, in **C:** 20 μm.

Our model for gene expression in the periproctodeal region implies that cells are cycling between expressing and non-expressing states. To examine this in more detail, we have carried out hybridizations with a probe from the *Delta* gene that contains a largely (>95%) intron sequence (Additional file 2: Figure S1) to monitor the distribution of nascent transcripts, and hence to reveal where transcription is most active (Figure 6). This probe reveals intense “nuclear dots” of nascent *Delta* transcripts in cells of the periproctodeal region, suggesting that *Delta* is strongly transcribed in this region. In the same embryos, the nuclear dots are much less frequent, and much less intense, in the resolved rings of *Delta* transcript accumulating further from the proctodeum. In these rings, the relatively weak hybridization of exon sequences in the probe to cytoplasmic transcript dominates the nuclear signal, suggesting that rates of transcription are much lower here. (Figure 6; see Additional file 3: Figure S2, Additional file 4: Figure S3 and Additional file 5: Figure S4 for similar preparations of older embryos). These observations are consistent with the proposal that expression in the periproctodeal region of the *Strigamia* embryo is highly dynamic, with transcripts being cleared from cells once every cycle of the pattern. In the resolved stripes, *Delta* transcripts may be more stable here than they are in the periproctodeal region.

In the presomitic mesoderm of zebrafish, dynamic waves of gene expression across the tissue are revealed by an offset between the distribution of nascent nuclear transcripts and the accumulation of cytoplasmic transcript for genes, such as *Delta*[17]. This offset is due to the delay in the appearance of cytoplasmic transcript relative to the onset of transcription, and hence depends in part on the length of the transcription unit. We have not observed convincing evidence for an offset between nascent and cytoplasmic *Delta* transcript, in either the peri-proctodeal region, or in the resolved stripes. The transcription unit of the *Delta* gene is only 8.3 kb long though, which, in the context of the relatively slow rate of *Strigamia* segmentation, may not generate a sufficient delay between the onset of transcription and the appearance of cytoplasmic transcript.

### Concentric proctodeal patterning and germ band formation are separable processes

**Figure 7:**
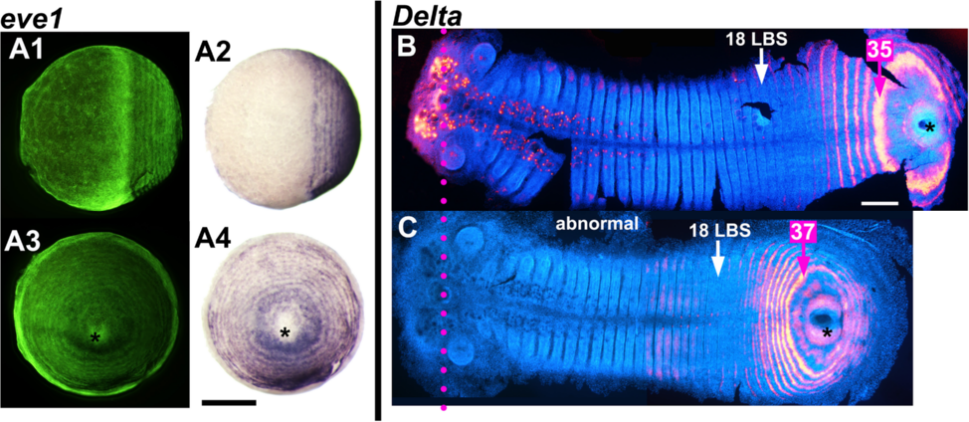
**Independence of concentric periodic patterning from germ band formation and elongation. A:** A spontaneous abnormal embryo showing primary concentric periodic patterning of the gene *eve1*, but not germ band formation. The same embryo is viewed laterally (**A1** and **A2**) and orthogonally to that view from the proctodeum (asterisk) (**A3** and **A4**) (**A1** and **A3** same view, respectively, as **A2** and **A4**, but under fluorescent light (SYBR green nuclear staining). **B-C:** A normal germ band **(B)** and an abnormally shorter one **(C)** with the same number of morphologically formed LBS (18), stained for *Delta*, flat mounted and aligned (magenta dotted line marking the stomodeum). In the short embryo, the number of rings of delta expression that have resolved is similar to that in the normal embryo at the same stage, but the spacing of these rings is very different. False colored *Delta* staining has been overlayed on the germ band viewed in fluorescent light to show the morphology (DAPI nuclear staining). Note that in **C** the artificial overlap has been done only for the posterior segmental patterning). Anterior *Delta* stain shows as dark dots. Asterisk marks the proctodeum. Scale bar in **A:** 400 μm; in **B** and **C:** 100 μm.

We have observed a rare but telling instance of a spontaneous abnormal embryo in which the process of concentric posterior patterning has proceeded in the absence of any germ band formation (Figure 7A). At least 10 rings of *eve1* expression are present, but there is no sign of germ band elongation/convergent extension and only very limited condensation, possibly of the precursor of the head. The embryo is apparently at a late blastoderm stage, but *eve1* expression is much more finely patterned than would be expected at this stage, and the embryo may in fact be much older. This appears to be a case where dorsoventral and/or axial patterning have failed, but concentric patterning has continued.

This exceptional embryo is one of a clutch where all other embryos showed defects in germ band elongation. Figure 7C depicts an embryo from another clutch, stained for *Delta*, which showed a similar defect. The germ band has formed, but is shorter and extends over less of the egg surface than in normal embryos with similar numbers of segments and primary rings of *eve* expression (Figure 7B). In these embryos segment patterning in the posterior disc seems to be compressed, and there are fewer cells between Delta stripes than in a normal embryo: four to five cells between primary stripes just before intercalation in the short embryo of Figure 7C, compared with seven to nine cells between stripes at the same position in a normal embryo (Figure 7B).

### Loss of oscillatory expression correlates with a late transition from double to single segment addition

The dynamic patterns described above are observed throughout the period of trunk segment patterning until the primary rings corresponding to about the 38^th^ leg-bearing segment have been generated (approximately the 26 LBS stage, beginning of stage 4.3).

**Figure 8:**
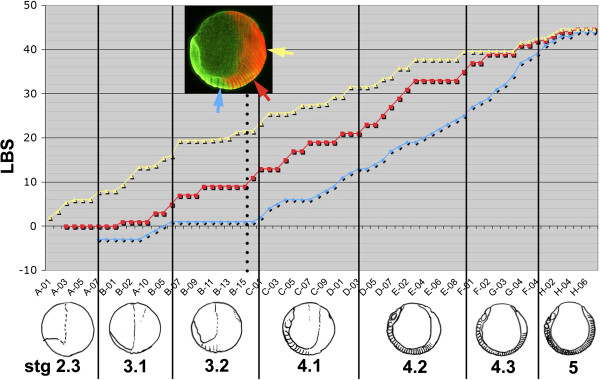
**Relative timing of appearance of primary double *eve1 *stripes, intercalating *eve1 *stripes, and morphological segments.** The plot shows the correlation among the appearance of morphological segments (blue), intercalated *eve1* stripes marking single segment periodicity (red) and primary *eve1* stripes of double segment periodicity (yellow). Each position along the X axis represents a single embryo from one of eight clutches (**A-H**, individual embryos numbered), ordered by the developmental age of each embryo, as defined by these markers. The location of the markers on one embryo (B15) is illustrated in the inset. The Y axis plots the number of leg-bearing segments (blue points) or the LBS number that each *eve1* stripe will eventually correspond to (red and yellow points). The numbering of primary stripes has been subdivided to indicate the extent of stripe resolution. For example, the embryos illustrated in Figure 4, panels A3, B3, C3, D3, which show the progressive resolution of primary stripe 28, would be assigned the values 26.0, 26.4 (the onset of cycle 28), 26.6 and 26.8. Developmental stages (stages 2.3 to 5), shown in schematic form below the plot, are delimited by vertical lines.

At the early stages of trunk segment patterning, the double segment pattern persists for about five repeats before the intercalation of segmental stripes. This implies that there is a considerable delay (about 30 hours at 13C) between the generation of the double segment pre-pattern, and the resolution of the single segment repeat. However, as segmentation progresses, the appearance of intercalating stripes “catches up” with primary ring formation. This is demonstrated in Figure 8, which plots, for a series of embryos ordered by age, the appearance of morphological segments (in blue), the activation of *eve1* in segmental intercalated stripes that presage the appearance of the odd numbered LBS (in red) and the first appearance of the double segment “primary stripes” that will resolve to define the even numbered LBS (in yellow). For example, for the embryo highlighted by the inset photo in Figure 8 (embryo B-15), only one leg-bearing segment is morphologically visible; *eve* segmental stripes are resolved up to the 9^th^ LBS but primary *eve* expression corresponding to the 22^nd^ leg-bearing segment is already appearing. However, the yellow and red lines approach one another progressively as segmentation proceeds, until in embryos with more than 30 leg-bearing segments, the two phases of *eve* expression are no longer distinct. *eve* and *Delta* stripes corresponding to later segments appear individually, with no double segment pre-patterning.

In most specimens of our population, the last resolved primary ring of *eve* that generates two segments is that corresponding to the 38^th^ or 40^th^ segment. For *Delta*, the last primary pair-rule ring is that corresponding to LBS 39 or 41. Some variability here probably reflects variation in the final segment number of the adult animals, which in our population ranges from 43 to 53, with the great majority of the male individuals having 47 segments, and the females 49 segments.

**Figure 9:**
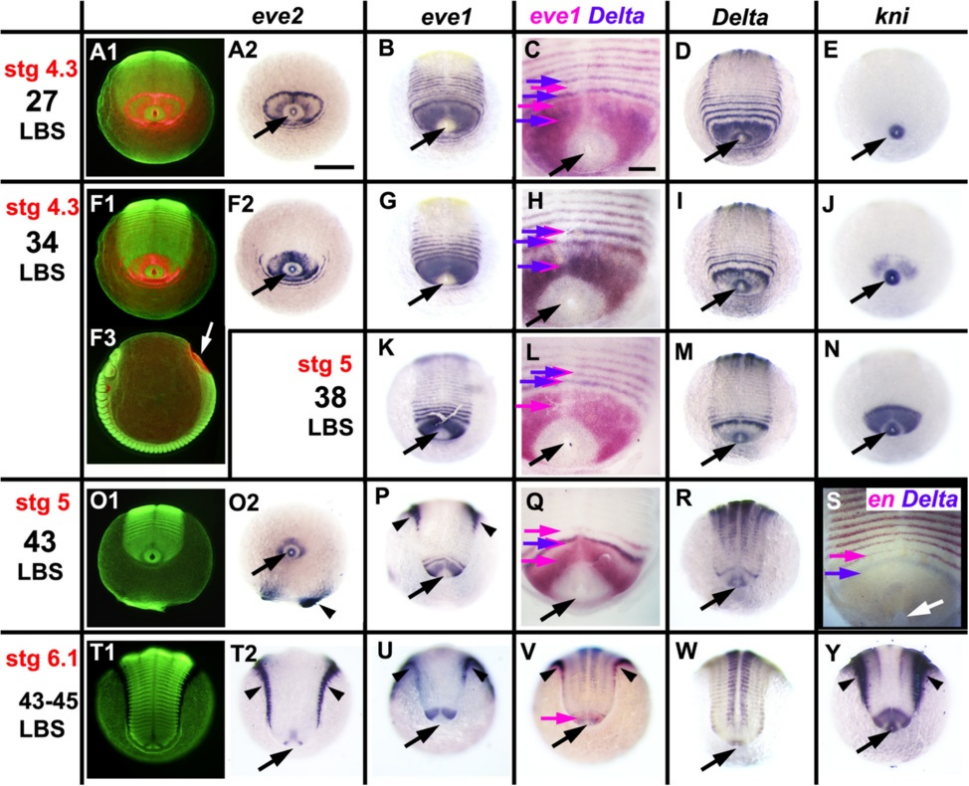
**Segmentation last phase: loss of oscillation and shift from double to single-segment periodicity.** Embryos are aligned as in Figure 1. In all but **F3**, the view is from the posterior pole - anterior on top. Columns 2 to 6 depict the expression of a single gene, or pair of genes, as indicated on top. Column 1 presents the same view as column 2, but under fluorescent light (SYBR green nuclear staining) **(A1, F1, O1, T1)**. In **A1**, **F1** and **F3***eve2* expression in red is overlaid on the nuclear staining. **F3** presents the same embryo as **F1**, but in lateral view, to clarify orientation for all embryos: *eve2*: Expression in primary “pair-rule” rings **(A2)** is lost around the 34 LBS stage **(F2, O2, T2)**. *eve1*: Stripe intercalation is still visible at the 27 LBS stage **(B)**. By the 34 LBS stage, all visible stripes are at single segment intervals **(G, K, P, U)**, as shown by the coincidence of *eve* and *Delta* staining **(H, L)**. The embryo in **P** is asymmetric, with one more stripe resolved on the left. *Delta*: Stripe intercalation is lost by the 43 LBS stage; the broader peri-proctodeal expression **(D, I)** is reduced **(M)**, to a single stripe at its anterior margin **(Q, R, V)**, eventually completely disappearing **(W)**. *engrailed* expression appears segmentally immediately anterior to the single *Delta* stripe **(S)**. *knirps***(E)** is up-regulated in the peri-proctodeal zone around the 34 LBS stage **(J)** and is persistently on thereafter **(N, Y)**. Black (white in **F3** and **S**) arrows: proctodeum; purple arrows: *Delta* expression; magenta arrows: *eve1* expression except in **S** where it marks *en* expression. Arrowheads: background staining commonly observed at the periphery of late germ bands. Scale bar in **C, H, L, Q** and **S**: 100 μm, in all other panels: 200 μm.

At this time, the pattern of gene expression in the peri-proctodeal area changes suggesting that this segmenting tissue has entered a new regulatory regime. From about the stage of embryo G-03 in the plot of Figure 8 (that is, 32 LBS visible), *eve1* is uniformly expressed in the peri-proctodeal area, with no apparent oscillation (Figure 9G, K, P U); the *eve2* gene, which only ever shows double segment periodicity, is progressively repressed (Figure 9F, O, T), with the expression fading most quickly from the germ band tissues (Figure 9 F2). At the anterior margin of the peri-proctodeal area, segmental *eve1* stripes resolve from the uniform domain of expression. These are limited to the germ band - they do not encircle the proctodeum. *Delta* is co-expressed with *eve* in these stripes (Figure 9H), in sharp contrast to the alternating expression that characterizes the earlier pair-rule activation of *Delta* and *eve* (for example, Figure 9C). By the time segment addition has paused at late stage 5, around the 43 LBS stage, *Delta* expression has become restricted to a single apparently stable band just posterior to the last formed segment (Figure 9Q, R) and only one or two *eve* stripes persist after resolution (Figure 9P, Q). By this time, the activation of *engrailed* expression occurs close to the *Delta* stripe, appearing just one segment’s width anterior to it (Figure 9S, Additional file 6: Figure S5). Eventually, segmental *Delta* expression fades completely just before the onset of germ band spreading in stage 6, although stable *Delta* expression persists in the proctodeal ring itself (Figure 9V, W). The lack of resolving *eve* stripes at stage 6 (Figure 9U) correlates with the pause in segment addition at this stage.

These changes also affect some genes that are not cyclically expressed - for example, *knirps. knirps* is expressed throughout the posterior of the egg at very early stages (C. Brena, unpublished), but during stage 4 it is repressed in all territory posterior to the second leg-bearing segment, except for the proctodeal ring itself (Figure 9E, J, N, Y). It is re-expressed specifically in the unsegmented territory anterior to the proctodeum around the 34 LBS stage, at about the time that oscillation stops (Figure 9J), and expression is maintained throughout stages 5 and 6 (Figure 9N, Y).

The rate of appearance of morphologically defined segments, which has been constant throughout stage 4 [14], continues unchanged for a period after the cessation of oscillation, while the remaining primary bands resolve from double to single segment periodicity (Figure 8, Additional file 6: Figure S5). However, the segments that are actually patterned from this time on appear much more slowly than those generated from the double segment prepattern: segmentation slows to a rate of about 1.6 segments per day, compared with 7.5 segments per day during stage 4 [14].

### The early onset of periodic patterning

Expression of the segmentation genes that we are considering here initiates during the blastoderm stage, while the head segments are being patterned. Our understanding of these earliest stages of segment patterning is less clear, in part because these early embryos are extremely difficult to preserve during the process of *in situ* hybridization. However, the data that we have show significant parallels with the later phase of trunk segment addition, though with some unique features.

A key question to address for these early stages is whether anterior segments are patterned by a process similar to that which occurs in the trunk, which seems to involve dynamic patterning of the pair-rule genes as the primary process, or whether they are patterned by a process more akin to that in *Drosophila*, where subdivision of the embryo occurs with respect to localized transcription factors of the gap gene type, which generate a non-periodic spatial pre-pattern that instructs the pair-rule pattern.

Below, we present the data that are available for *eve* and *Delta* gene expression during these early stages, before addressing this question explicitly in the Discussion.

#### even-skipped

**Figure 10:**
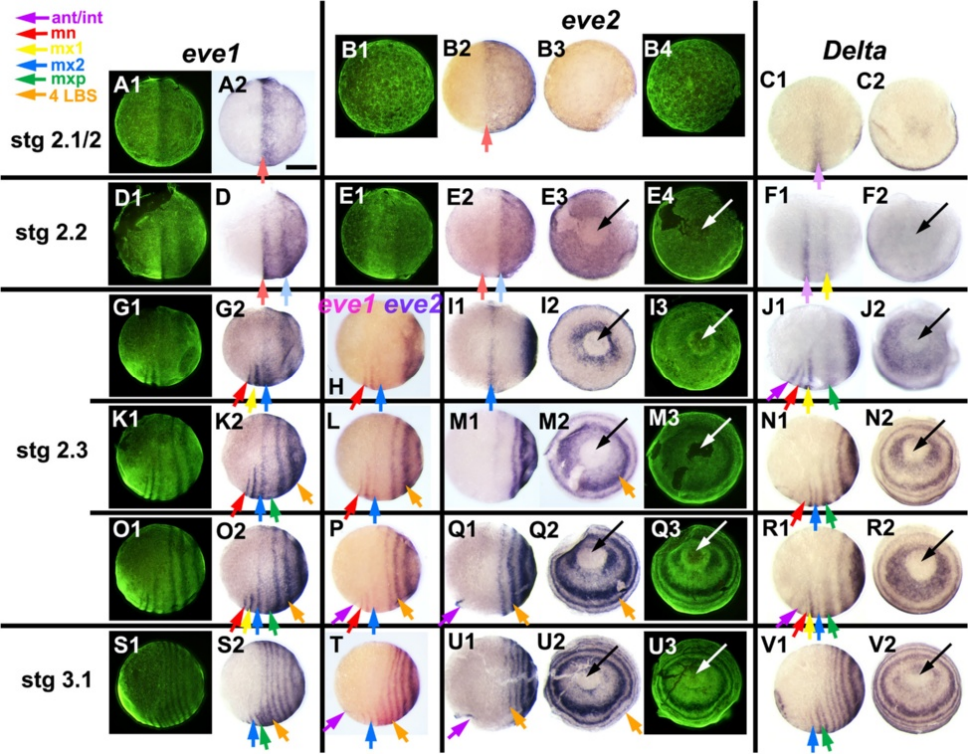
**Expression of *even-skipped *and *Delta *genes at the onset of periodic patterning.** Embryos are aligned as in Figure 1, from blastoderm stage 2.1, to the appearance of the first morphological segment, the mandible, at early stage 3.1. Panels with the same letter present different views of the same embryo (either lateral, or a view from the posterior pole orthogonal to these lateral views, with the blastopore/proctodeum at the center (black or white arrow). Ventral is to the bottom in all images. Fluorescent nuclear staining (SYBR green) for the embryos hybridized with *eve1* (lateral view) and *eve2* (posterior view) represents the morphology for the whole row. The temporal alignment of embryos within rows is based on double *in situ* for *eve1* and *eve2***(H**, **L**, **P**, **T)** and for *eve1* and *Delta* (data not shown). Note that embryo **B** is younger than embryos **A** and **C**. The entire pattern is dynamic at this stage, condensing anteriorly as the pattern resolves. Based on a larger series of embryos (see Additional file 7: Figure S6), we have inferred continuity of expression between stages, as for example in the comparison of **K2** and **O2** in this plate. Expression domains are shown in this figure with arrows corresponding to the identity of the morphological segments eventually associated with each stripe, color-coded as indicated. A lighter color has been used for stages 2.1 and 2.2, where this identification is uncertain. (ant/int - antennal/intercalary segment (apparently deriving from same initial stripe); mn, mandible; mx1, first maxilla; mx2, second maxilla; mxp, maxilliped; 4LBS, fourth leg-bearing segment). Note that in **N1**, **R1**, **V1**, the green arrow marks *Delta* expression associated with a single stripe that splits to give rise to both the maxilliped and the first LBS. Scale bar: 400 μm.

At the uniform blastoderm stage (Stage 2.1, Figure 10), the *even-skipped* genes are expressed in a broad posterior domain that occupies the whole of the posterior hemisphere of the egg. The few embryos that we have at early blastoderm stages suggest that this expression initiates at the posterior pole of the egg and spreads anteriorly as a growing cap (Figure 10B; *eve2* staining).

As the head primordium begins to condense ventrally, and to move anteriorly, the uniform expression of *eve* becomes modulated by the first signs of segment patterning. For *eve1*, a single broad anterior stripe resolves at the anterior of the expression domain, near the equator of the egg (Figure 10A, D). Within the territory of the head condensation, this quickly resolves into two segmental stripes that define the mandibular and first maxillary segments (Figure 10G; Note that this figure presents only a subset of the embryos used to assign segment identities to bands; for *eve1*, the complete series of available embryos is shown in Additional file 7: Figure S6.). Outside of the germ band, *eve1* expression fades completely. A second broad stripe resolves behind the first, initially at 25 to 30% egg length from the posterior pole (Figure 10D), but moving forwards until it too lies near the equator, close behind the now resolved mandibular and first maxillary stripes (Figure 10G). Further stripes resolve more posteriorly, defining the double segment periodicity that will characterize *eve* expression throughout later development (Figure 10G, K, O, S).

*eve2* shows a similar pattern of primary stripe formation, but expression fades completely without ever resolving to single segment periodicity. The transcript disappears slightly faster, if anything, from the germ band than from the surrounding territory. The most anterior stripe is barely resolved before fading (Figure 10E); the second stripe persists slightly longer, becoming well resolved as it moves forward towards the middle of the egg (Figure 10I), before fading as it reaches about 60% egg length (Figure 10M).

As the second stripe fades, posterior expression of *eve2* becomes up-regulated immediately around the blastopore, which is now sharply defined as an area free of *eve* expression (Figure 10E). A series of closely staged eggs suggest that this expression spreads until it uniformly fills a posterior cap that extends to about 30% egg length. A third *eve* stripe then resolves from the margin of this cap. The process repeats, as trunk segmentation becomes established, with each new stripe fading shortly after it is formed (Figure 10M, Q, U). (Later, a single transient stripe of *eve2* expression arises in the antennal segment, but the relation of this to early *eve2* expression is unclear; Figure 10P-Q, T-U).

#### Delta

During the uniform blastoderm stage, while the *eve* genes are expressed as a posterior cap, *Delta* is expressed as a single broad stripe around the ventral two-thirds of the egg’s circumference, at 50% egg length. There is also a faint patch of *Delta* expression at the posterior pole (Figure 10C). The ventral stripe appears to move forward until it lies at about 60% egg length, when a second stripe appears behind it (Figure 10F). It is not clear whether this second stripe is ever preceded by broad expression of *Delta* in the posterior hemisphere.

At around the onset of head condensation, these two anterior *Delta* stripes resolve into a pair of major stripes coincident with the *engrailed* stripes of the intercalary and first maxillary segments, and a minor stripe of the intervening mandibular segment (Figure 10J). At the same time, posterior expression of *Delta* appears, first as a uniform posterior cap, and then resolving to a series of primary bands. Like *evenskipped*, *Delta* expression is quickly excluded from the region of the blastopore itself, but each new primary band arises with strong expression around the blastopore (Figure 10J, N, R, V). The primary bands of *Delta* arising from the posterior cap mark the first, third, fifth and successive leg-bearing segments. Exceptionally, the first of these primary *Delta* bands appears to split to give rise to both the maxillopedal and the first leg-bearing segment (Figure 10R, V). Thereafter, secondary stripes intercalate to mark the second, fourth and successive even-numbered LBS.

## Discussion

There are currently two models for the generation of segments in arthropods. In the *Drosophila* paradigm, initially graded signals lead to the localized activation of the segment “gap genes”, encoding transcription factors that provide spatial cues unique to each position along the anteroposterior axis. These cues instruct periodic activation of segmentation genes using distinct transcriptional circuitry for different segments. We will refer to this as a “gap gene” based model.

The other model involves a cyclic, dynamic process of gene expression analogous to that which has been analyzed in some detail for vertebrate somitogenesis [5,18]. Such a model has been proposed as the mechanism for segment formation in short germ arthropods, and is supported by experimental data in a number of species [19-22], although these data are open to alternative interpretations [23,24]. However, two recent papers provide clear evidence for cyclic waves of gene expression preceding segmentation in the short germ beetle *Tribolium*[3,4]. We will refer to this second class of model as a clock-based process.

### A clock-like mechanism in *Strigamia*

The evidence supporting a clock-like oscillation of eve and *Delta* expression in the peri-proctodeal area of *Strigamia* can be summarized as follows:

– the coherent transformation of the observed expression patterns in embryos placed into a closely staged developmental series by other criteria (Figure 4).

– the rapid dynamics of change in the peri-proctodeal area, contrasting with the relative stability of the surrounding resolved pattern (Figures 4E and 5A, B).

– the observation of asymmetric patterns in single embryos that closely resemble different stages of the inferred cycle (Figures 4F and 5C).

– the extent of the egg surface that shows dynamic expression in the earliest stages of patterning (Figure 10), which appears to rule out evolution of these patterns by wholesale cell movement.

This evidence falls short of that which could be provided by embryo culture experiments coupled with cell marking, but such techniques have not yet proved possible in *Strigamia*. However, the available data make a strong argument that the patterns of expression observed for *even-skipped* and *Delta* reflect repeated cycles of transcription within this whole cell population. It is likely that the similar patterns shown by homologues of the pair rule genes *hairy*, *runt* and *oddskipped* reflect similar dynamic patterns [11-13] (C. Brena, data not shown).

This oscillatory activity is apparent during the main phase of trunk segmentation. For *eve2* and *Delta* (at least), each cycle initiates at paired lateral sites adjacent to the proctodeum, once in each double segment cycle. This activation spreads, somewhat unevenly, throughout the majority of the peri-proctodeal area. Transcript is then cleared from the inner part of this area, leaving an ill-defined ring that sharpens and becomes more regular as it expands somewhat further.

The dynamic nature of this pattern is particularly clear during early stages of the segmentation process, when the primary rings that will give rise to the mouthparts and the most anterior leg-bearing segments are generated. During these earliest cycles, transcription of *eve* is activated throughout almost the entire posterior hemisphere of the egg excepting the area of the blastopore itself, and then cleared from all of this territory except an anterior marginal ring. Given that the cell density remains broadly uniform across the posterior hemisphere of the egg (which at this stage is the multilayered blastoderm [14], it is not feasible that the loss of expression from the posterior territory in each cycle is due to movement of all of these cells into the marginal ring.

The pacemaker for these oscillations seems to lie at or adjacent to the blastopore throughout the segmentation process. Initially, new cycles of gene expression appear concentrically around the extended blastopore, but as the morphology of the proctodeum develops, the initiation becomes bilateral, with new cycles of expression initiating at foci that appear as two patches lying antero-laterally just outside the proctodeal ring; tissues of the proctodeum itself do not show oscillating gene expression. This posterior territory is already multilayered [14], and clearly divided into a number of populations expressing distinct cell markers (data not shown). Oscillatory expression of the pair rule genes is restricted to the outer, ectodermal cell layer [13] at least by the 15 to 20 LBS stage. The observation that this oscillation can be maintained in an embryo that lacks any obvious sign of germ band formation (Figure 7A) suggests that axial patterning is distinct from the generation of periodicity that underlies segmentation.

It appears that gene expression becomes stable in individual cells at or shortly after the time that each ring becomes well resolved. The further slow expansion of this primary pattern reflects tissue movement driven by continuing proliferation of the posterior disc, and convergent extension of the forming segments as the whole egg epithelium condenses anteriorly and cells move into the germ band. However, we cannot at present say precisely where the transition from oscillatory to stable gene expression occurs.

### The prevalence of clock-like segmentation processes in arthropods

The oscillation of *eve* and *Delta* expression that we infer in *Strigamia* is similar to that recently reported for *odd-skipped* and *eve* in *Tribolium*, by Sarrazin *et al*. [3] and El-Sherif *et al*. [4]. These authors have shown by culturing bisected embryos, by comparing series of closely staged embryos and by tracking cell movements in the posterior of the germ band that the early stripes of *odd-skipped* and *eve* expression are indeed waves of oscillatory gene expression moving across cells. These waves are of double segment periodicity, and move in register but out of phase.

In no other arthropod has oscillatory gene expression been documented unambiguously, but the phenomenology of gene expression suggests that it exists quite widely. It was first suggested for opisthosomal segment patterning in spiders [19,20], and has been proposed for abdominal segment addition in at least one hemimetabolous short germ insect [21,25]. The concentric expression of segmentation genes around the proctodeum in the millipede *Glomeris* appears strikingly similar to what we observe in *Strigamia*[26]. Concentric patterns have been observed, at least for *even-skipped*, in another centipede, *Lithobius*[7]. However, in all of these cases the patterns of gene expression define a single segment periodicity, rather than the double segment periodicity observed in *Strigamia* and in *Tribolium*. This raises some question as to the homology of the oscillatory mechanisms that may exist across the arthropods. However, it may be that the processes which generate primary single segmental stripes in *Glomeris*, and perhaps other arthropods, may be homologous to the mechanisms that generate primary pair-rule patterning in *Strigamia*.

Dynamic oscillation of gene expression also occurs in the pre-somitic mesoderm of vertebrates, where the oscillatory mechanism has been analyzed in some detail [5]. The precise mechanism of the oscillator appears to vary between species [27], but in all vertebrates examined, the oscillation is of single somite periodicity, and is limited to the mesoderm, whereas in arthropods primary segment patterning involves the ectoderm. In zebrafish (at least) the oscillation seems to be a cell autonomous process, and the role of Notch signaling is to co-ordinate the phase of oscillation between cells [28]. The extent to which similar mechanisms underlie oscillatory gene expression in vertebrates and arthropods remains to be documented: modeling suggests that it may be rather easy to generate either (or both) spatial and temporal oscillation from gene networks that generate pattern [29]. Intriguingly, a cell autonomous circuit of pair-rule gene interaction has been described in *Tribolium*[30] but this might serve either to generate dynamic behavior, or to ensure that cell states are mutually exclusive once pair-rule periodic expression is established.

### Anterior segment patterning - clock-like or gap-gene based?

In even the most extreme of short germ insects, the segments of the mouthparts and anterior thorax appear to be patterned from cells generated during cleavage. Most authors have assumed that these segments in all insects are patterned by a gap-like mechanism dependent on localized factors upstream of the pair-rule genes [31] and we thought it likely that the same would be true in *Strigamia*. We therefore expected to see that the dynamic patterning of *Delta* in association with *eve* would initiate posterior to the first formed segments, approximately at the position of the first leg-bearing segment.

Our data confirm that segments of the trunk, from the second leg-bearing segment backwards, do appear to be generated by a simple repeat of the same cycle of gene expression states. However, to our surprise, both *Delta* and *eve* show patterns of dynamic expression that initiate at the very onset of segment patterning, and relate to the generation of pattern from the intercalary segment back.

For *eve*, this early expression takes the form of an initial activation throughout the entire posterior hemisphere of the egg, and then the resolution of stripes within this domain. This is reminiscent of early *eve* expression in insects, the only other arthropods that have been studied at these early stages. In *Drosophila*, the resolution of stripes within this early domain depends on the regulation of *eve* by gap genes.

It is possible that for *Strigamia* too, the early resolution of the *eve* pattern depends on upstream “gap like” factors. Gap gene domains themselves may shift dynamically due to gene interactions, and the expression of dependent pair-rule genes then follows them, as has been documented in the dipteran insect *Clogmia albipunctata*[32]. However, the observation that the dynamics of *Delta* parallel those of *even-skipped*, even for these very first stripes, and that the whole early pattern appears to move so dynamically across the blastoderm as it resolves, raises the question as to whether the patterning of even the most anterior segments, the intercalary and mouthpart segments, might depend on signaling interactions similar to those inferred for the more posterior trunk segments.

### The origins and significance of pair-rule patterning

Pair-rule patterning during segmentation was first described in *Drosophila*, where the mechanism is relatively well understood. In most other insects studied, at least some homologues of the *Drosophila* pair-rule genes are expressed with a double segment periodicity, although the function of some genes appears to differ between species. No pair-rule expression has been reported for segmentation genes in some short germ insects [33,34], in the few crustaceans studied [35,36], or for most segments in spiders [37], though in the latter the most anterior stripes of gene expression are involved in a splitting phase [38,39]. There is one report of pair-rule patterning for a *paired* (Pax3/7) homologue in the prosoma of a mite [40].

The pair-rule nature of primary segment patterning in *Strigamia* is very clear. However, no such pair-rule patterning is apparent in the lithobiomorph centipede *Lithobius atkinsoni*, where *even-skipped* expression has been studied in relation to segment polarity marker genes [7]. There is also no trace of pair-rule patterning during trunk segment patterning in the millipede, *Glomeris marginata*[26], although stripe splitting is observed in the head [41]. Thus, among the myriapods, pair-rule patterning of trunk segments has been observed uniquely in *Strigamia*. It may, therefore, be a feature that has evolved within centipedes, and may prove to be unique to geophilomorphs, or to the single derived lineage leading to geophilomorphs and scolopendromorphs [42,43], which is the clade of centipedes that shows the highest segment numbers. *Lithobius*, showing single segment addition, lies basal to the origin of this clade.

### The final phase of segment addition - from pair-rule to single segment periodicity

The transition from double to direct single segment patterning appears to be accompanied by a broad change in the regulatory regime occurring in the unsegmented posterior tissue: *eve1* expression becomes stable, and *eve2* and *Delta* expression are switched off in the posterior tissue at the time of this transition. At least one other transcription factor not previously active during the main phase of segmentation, *knirps*, is switched on. In contrast to the earlier phase of patterning, segmental stripes resolve only in the region of the germ band, and not around the entire periphery of the peri-proctodeal area. There is no indication that the whole tissue is undergoing dynamic cycles of gene expression.

This final phase of single segment addition undermines the argument we have made previously [11], that pair rule patterning might explain an observed constraint on the number of segments in centipedes. All centipedes have an odd number of pairs of leg-bearing segments as adults and, hence, an even number of trunk segments including the poison claw segment. This constraint is particularly striking for the geophilomorph centipedes, which show great inter- and intra-specific variability in the number of leg-bearing segments, which ranges from 27 to 191 [43].

This observation is readily explained as a developmental constraint if all trunk segments are generated in pairs. However, if the last segments are generated singly, we can only preserve this explanation by proposing that there is no variation in the number of singly patterned segments, and that the variation available within populations is restricted to the phase of double segment patterning. In this context, it is relevant that *Lithobius*, which appears to make its trunk segments singly [7], belongs to a clade that indeed shows no variation in segment number: all lithobiomorph centipedes have 15 pairs of leg-bearing segments. Invariant segment number is in fact frequent in all major arthropod lineages, particularly among relatively short bodied forms (for example, of no more than 20 segments). Thus it is not unreasonable to propose that, in the long bodied geophilomorphs, the variable generation of segments by pair rule patterning has been imposed on an invariant underlying body plan.

## Conclusions

The dynamic appearance of gene expression during segment patterning in *Strigamia* reflects two processes - an intrinsically dynamic process of gene expression in cells around the proctodeum, and the overall movement of cells in the germ band more anteriorly. A pair-rule segmentation oscillator underlies the generation of all anterior trunk segments, and may also be involved in patterning of head segments from the intercalary back. However, there appears to be a transition in the dynamics of segment addition at stage 4.3, such that oscillatory gene expression is suppressed, and the last 10 trunk segments are patterned individually, without a pair-rule pre-pattern. These alternative modes of behavior for the segmentation gene network in one species suggest that the different modes of segmentation observed in different arthropods may evolve rather readily from a largely conserved underlying gene network.

## Methods

*Strigamia maritima* eggs were collected, cultured, fixed, stained and photographed as described in [14].

For the preparation of *in situ* hybridization probes, previously cloned fragments of the *S. maritima* genes *eve1*, *eve2*[12]*knirps* (NCBI Accession: EF175909.1 to EF175912.1) and *dpp* (A. Chipman, unpublished data) were extended with gene-specific primers through 3′ and 5′ rapid amplification of cDNA ends (RACE) (BD **SMART**™ RACE cDNA Amplification Kit, Clontech, Mountain View, CA, USA). For *en* and *Delta* published clones were used to make the probes [12,15]. Probes for exon sequences were prepared using the longest available cDNA clone of each gene (2.2 kb for *dpp*, 1.3 kb for *knirps*, 0.7 kb for *en*; for *Delta*, *eve1* and *eve2,* see Additional file 2: Figure S1). The intron probes for *Delta* and *eve1* were designed as reported in Additional file 2: Figure S1, after mapping cDNA data against genomic scaffolds from the Smar_1.0 genome assembly [44]. The *Delta* ‘intron’ probe contains >95% intron sequence, but does span two short (60 bp) exons in the 2.8 kb sequence, and so detects cytoplasmic transcript weakly, as well as strong nuclear signal from nascent transcript. The *eve1* intron probe is derived purely from intron; this probe yielded no signal following *in situ* hybridization (see Additional file 2: Figure S1).

*In situ* hybridization was carried out essentially as described by [15,45], but in general with the anti-DIG antibody (Roche, Mannheim, Germany) at 1:3,000 dilution. In the case of intron probes, hybridization and staining reaction times have been increased to up to 20 h and 8 h, respectively, (in the case of the unsuccessful *eve1* intron probes to up to 40 h and 13 h, respectively).

Stages of embryos were estimated as described in [14]. During stages 3 to 5, embryos were further staged by recording the number of the last leg-bearing segment morphologically visible, that is, defined by both anterior and posterior segmental grooves.

## Abbreviations

DIG: Digoxigenin; dpp: decapentaplegic; en: *engrailed*; eve: *even-skipped*; LBS: Leg bearing segment.

## Competing interests

The authors declare that they have no competing interests.

## Authors’ contributions

CB carried out all of the experimental work, recognized the transition from double to single segment patterning, and developed the first interpretation of the data. MA participated in the interpretation of the data, and both authors wrote the manuscript. Both authors read and approved the final manuscript.

## Supplementary Material

Additional file 1: Movie 1 A single oscillation of *eve1* expression. Possible representation of a single oscillation of *eve1* expression, obtained by overlapping ordered embryos **A-D** of Figure 4 (younger embryo on top), with increasing transparency of the younger embryo in each sequential frame. The ring corresponding to the 24^th^ LBS (marked by red arrows), external to the dynamic peri-proctodeal area, shows no detectable movement over this time window. Click here for file

Additional file 2: Figure S1 Gene structure for the genes *Delta*, *eve1* and *eve2*. Gene structure and extension of *in situ* probes for the genes *Delta*, *eve1* and *eve2*, mapped against genomic scaffolds from the Smar_1.0 genome assembly ( http://www.ncbi.nlm.nih.gov/assembly/322118/), and gene models derived from the current genome annotation (pending submission to Ensembl Metazoa - web link to be provided at proof stage(, which incorporates RNAseq data as well as the cDNA information. The genome map is represented on a light blue background. Bars represent exons, the blue-filled parts being the coding regions, and the open parts the UTRs; 5′ on the left; red lines mark the stop codons. Hybridization *in situ* probes are represented on a black background, with exon probes in yellow (derived from cDNA clones) and intron probes in light blue, derived from genomic DNA: note that in the case of *Delta* the two intron probes each include a small exon (60 nucleotides). The two exclusively intronic probes of *eve1* have not yielded any detectable signal on *in situ* hybridization. (Data from *Strigamia maritima* genome assembly Smar_1.0. Note that the three gene maps are not to the same scale. Click here for file

Additional file 3: Figure S2 Distribution of *Delta* nascent transcripts in a late stage 3 embryo. A1 Flat mount of an embryo at the stage with one leg-bearing segment morphologically visible (1LBS stage), hybridized with a *Delta* probe comprising approximately 95% intron and 5% exon sequence (see Additional file 2: Figure S1). This probe detects unprocessed nascent transcripts much more strongly than spliced cytoplasmic transcripts. At this early stage there are prominent nuclear dots of nascent transcript (magenta arrowheads) in many cells of the peri-proctodeal region (enlarged in panel A3); double nuclear dots are often visible, showing transcription associated with both copies of the gene. In more mature *Delta* stripes, transcript is still present at high level in the cytoplasm, but nuclear dots are less frequent and less prominent (enlarged in A2). Note that embryos at this stage extend around much of the egg, and are therefore distorted and split at the lateral margins when flattened. The head is to the left. Black arrowhead: mandibular segment an asterisk marks the proctodeum. Scale bar in A1: 200 μm, in A2-3: 20 μm. Click here for file

Additional file 4: Figure S3 Distribution of *Delta* nascent transcripts in an early stage 4 embryo. A1 Flat mount of an embryo at the 15 LBS stage, hybridized with a *Delta* probe that detects unprocessed nascent transcripts much more strongly than spliced cytoplasmic transcripts (see Additional file 2: Figure S1). At this stage nuclear dots of nascent transcript (magenta arrowheads) are still prominent in many cells of the peri-proctodeal region (enlarged in A4, A5), although the signal intensity may be somewhat lower than in earlier stages. In more mature *Delta* stripes, transcript is still present at high level in the cytoplasm, but nuclear dots are less frequent (insert A3). Nuclear dots are almost undetectable in the oldest, more anterior stripes (insert A2). The head is to the left. Black arrowhead: mandibular segment; an asterisk marks the proctodeum. Scale bar in A1: 200 μm, in A2-5: 20 μm. Click here for file

Additional file 5: Figure S4 Distribution of *Delta* nascent transcripts in an early stage 5 embryo. A1 Flat mount of an embryo at the 41 LBS stage, hybridized with a *Delta* probe that detects unprocessed nascent transcripts much more strongly than spliced cytoplasmic transcripts (see Additional file 2: Figure S1). At this stage *Delta* transcripts are no longer detectable in the peri-proctodeal region. Two posterior stripes of *Delta* expression remain, defined by an accumulation of cytoplasmic transcript, but nuclear dots of nascent transcript are no longer detectable in these stripes (insert A4). Nuclear dots show that transcription of *Delta* is activated at high level in more anterior medial cells (magenta arrowheads in insert A3). These cells are neuronal precursors. In more mature segments they will form clusters where *Delta* is present at high level in both the nuclei and the cytoplasm (insert A2). The head is to the left. Black arrowhead: mandibular segment; an asterisk marks the proctodeum. Scale bar in A1: 200 μm, in A2-4: 20 μm. Click here for file

Additional file 6: Figure S5 Last phase of segmentation: from double (primary *Delta*) to single-segment periodicity (*engrailed* and secondary *Delta*). In a 34 LBS embryo **(A)***Delta* still appears at double segment periodicity (large blue arrowheads); intercalated *Delta* stripes defining the single segment periodicity appear only slightly later, that is, more anteriorly, as the segment pattern matures (small blue arrowheads). *engrailed* expression (magenta arrowheads) appears yet slightly later than intercalated Delta, overlapping with the segmental stripes of Delta as these fade. *engrailed* is then expressed persistently in the whole maturing germ band anterior of the segment addition zone. In a 36 LBS embryo **(B)**, the single segment intercalation of *Delta* appears just after the earliest resolved *Delta* band; *engrailed* expression appears closer to this first *Delta* stripe. By the 43 LBS stage **(C)**, only a single stripe of *Delta* transcript persists. The transcription of engrailed initiates at a position just one segment anterior to this *Delta* stripe, but *Delta* expression has already faded from this anterior region. Scale bar in A1, B1, C1: 100 μm, in A2-C2: 50 μm. Click here for file

Additional file 7: Figure S6 The complete set of 28 embryos used to deduce the maturing pattern of *eve1* expression as presented in main text Figure 10. These embryos have been put into a developmental series using both morphological staging markers and the transitions in the *eve* pattern itself. Embryos A, D, G, K, O and S are the embryos indicated with the same letters in Figure 10, selected from this full series. The correspondence of bands at the different stages was inferred with reference to the full series, allowing for example the conclusion that the Mx2 band (blue arrow in Figure 10, red arrow in Additional file 7: Figure S6) moves anteriorly during stages 2 and 3. Similar, though less extensive, series were used to infer the correspondence of Delta and eve2 expression patterns between stages. Each embryo is presented in a single row. Columns 1 and 2 - lateral views; columns 2 and 3 - ventral views; columns 4 and 5 posterior views; paired nuclear fluorescence and bright field images. Click here for file
