# Supplementary figures and images for: An analysis of segmentation dynamics throughout embryogenesis in the centipede Strigamia maritima
Source: BMC Biol. 2013 Nov 29;11:112. doi: 10.1186/1741-7007-11-112 (PMC3879059; doi:10.1186/1741-7007-11-112)

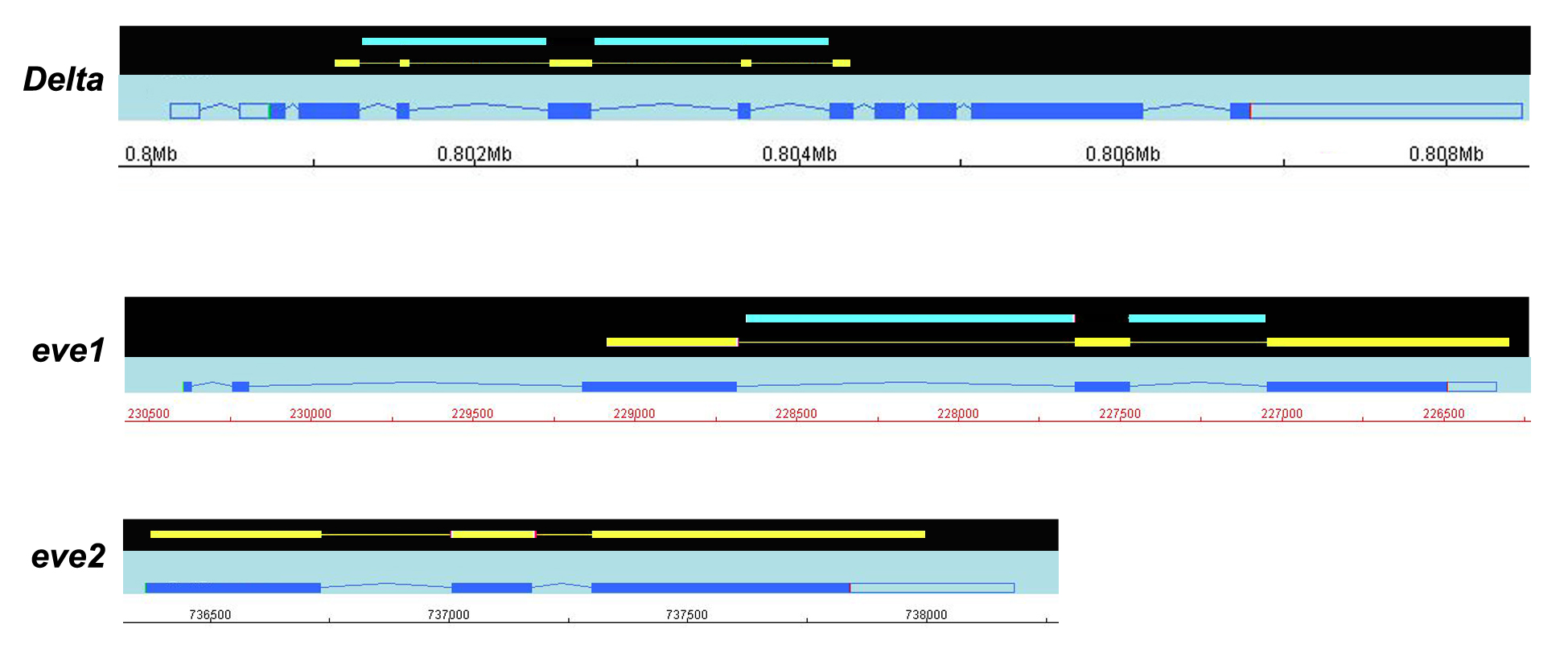

Supplement: Additional file 2: Figure S1 — Gene structure for the genes Delta, eve1 and eve2. Gene structure and extension of in situ probes for the genes Delta, eve1 and eve2, mapped against genomic scaffolds from the Smar_1.0 genome assembly ( http://www.ncbi.nlm.nih.gov/assembly/322118/), and gene models derived from the current genome annotation (pending submission to Ensembl Metazoa - web link to be provided at proof stage(, which incorporates RNAseq data as well as the cDNA information. The genome map is represented on a light blue background. Bars represent exons, the blue-filled parts being the coding regions, and the open parts the UTRs; 5′ on the left; red lines mark the stop codons. Hybridization in situ probes are represented on a black background, with exon probes in yellow (derived from cDNA clones) and intron probes in light blue, derived from genomic DNA: note that in the case of Delta the two intron probes each include a small exon (60 nucleotides). The two exclusively intronic probes of eve1 have not yielded any detectable signal on in situ hybridization. (Data from Strigamia maritima genome assembly Smar_1.0. Note that the three gene maps are not to the same scale. [file 1741-7007-11-112-S2.jpeg]

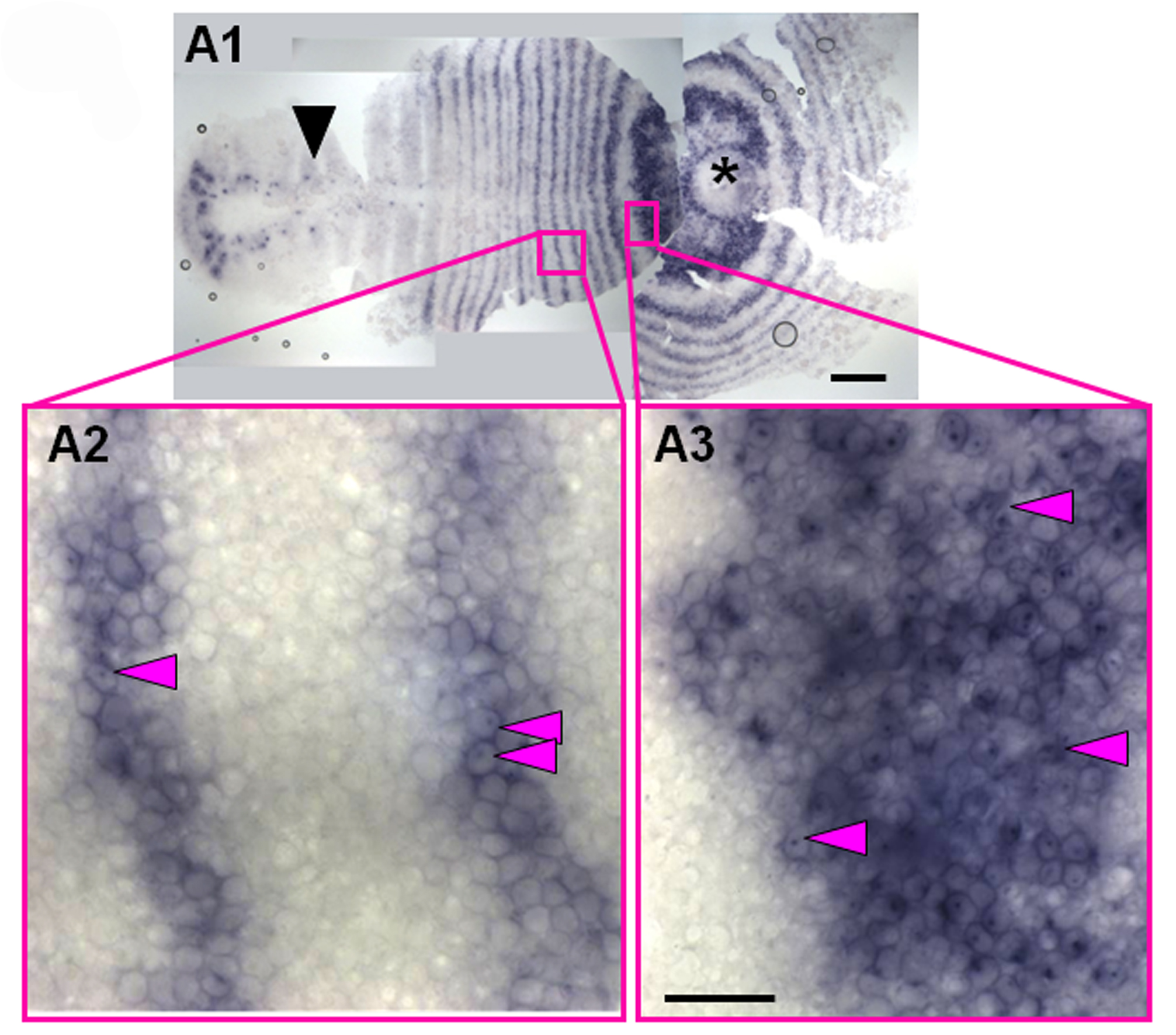

Supplement: Additional file 3: Figure S2 — Distribution of Delta nascent transcripts in a late stage 3 embryo. A1 Flat mount of an embryo at the stage with one leg-bearing segment morphologically visible (1LBS stage), hybridized with a Delta probe comprising approximately 95% intron and 5% exon sequence (see Additional file 2: Figure S1). This probe detects unprocessed nascent transcripts much more strongly than spliced cytoplasmic transcripts. At this early stage there are prominent nuclear dots of nascent transcript (magenta arrowheads) in many cells of the peri-proctodeal region (enlarged in panel A3); double nuclear dots are often visible, showing transcription associated with both copies of the gene. In more mature Delta stripes, transcript is still present at high level in the cytoplasm, but nuclear dots are less frequent and less prominent (enlarged in A2). Note that embryos at this stage extend around much of the egg, and are therefore distorted and split at the lateral margins when flattened. The head is to the left. Black arrowhead: mandibular segment an asterisk marks the proctodeum. Scale bar in A1: 200 μm, in A2-3: 20 μm. [file 1741-7007-11-112-S3.jpeg]

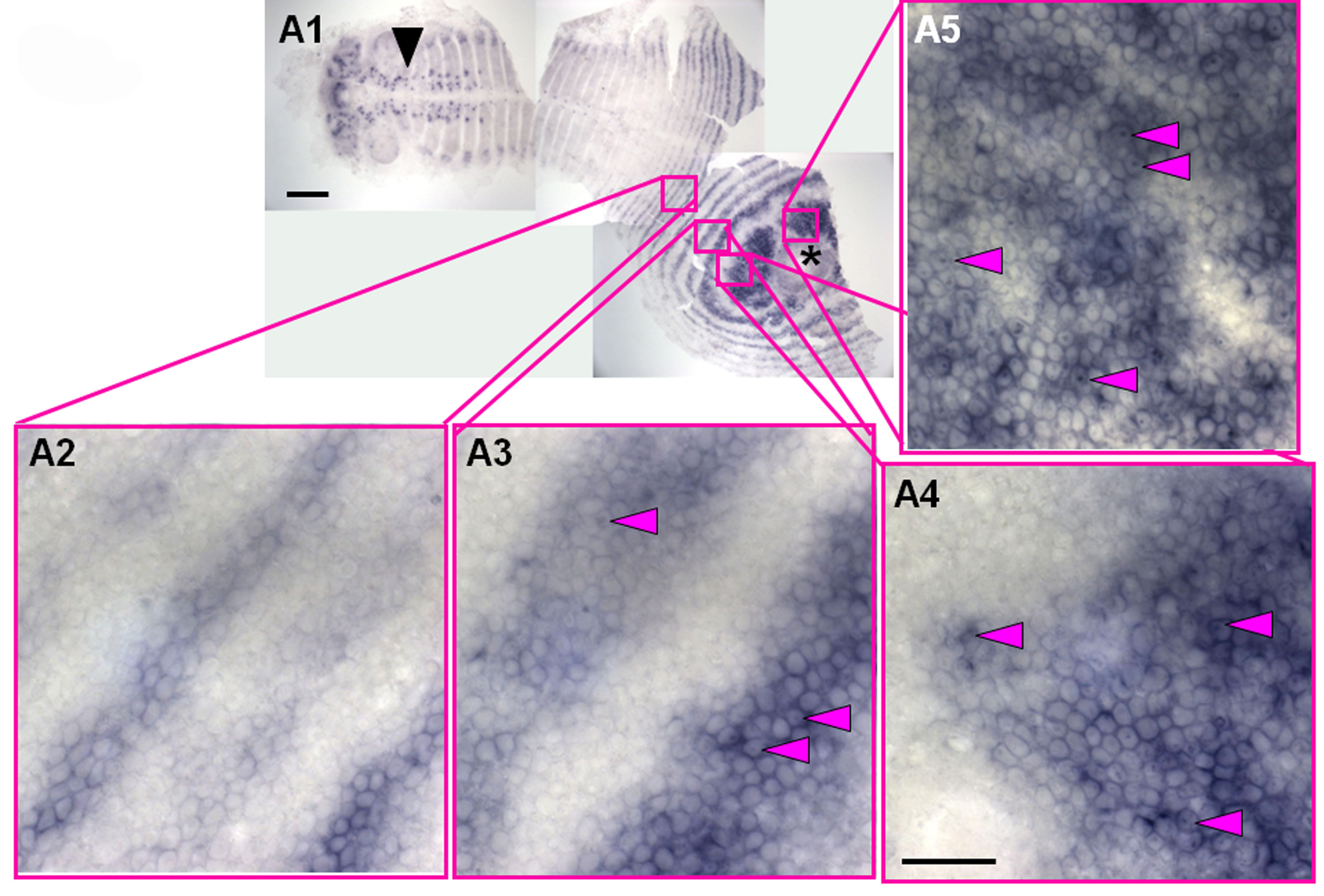

Supplement: Additional file 4: Figure S3 — Distribution of Delta nascent transcripts in an early stage 4 embryo. A1 Flat mount of an embryo at the 15 LBS stage, hybridized with a Delta probe that detects unprocessed nascent transcripts much more strongly than spliced cytoplasmic transcripts (see Additional file 2: Figure S1). At this stage nuclear dots of nascent transcript (magenta arrowheads) are still prominent in many cells of the peri-proctodeal region (enlarged in A4, A5), although the signal intensity may be somewhat lower than in earlier stages. In more mature Delta stripes, transcript is still present at high level in the cytoplasm, but nuclear dots are less frequent (insert A3). Nuclear dots are almost undetectable in the oldest, more anterior stripes (insert A2). The head is to the left. Black arrowhead: mandibular segment; an asterisk marks the proctodeum. Scale bar in A1: 200 μm, in A2-5: 20 μm. [file 1741-7007-11-112-S4.jpeg]

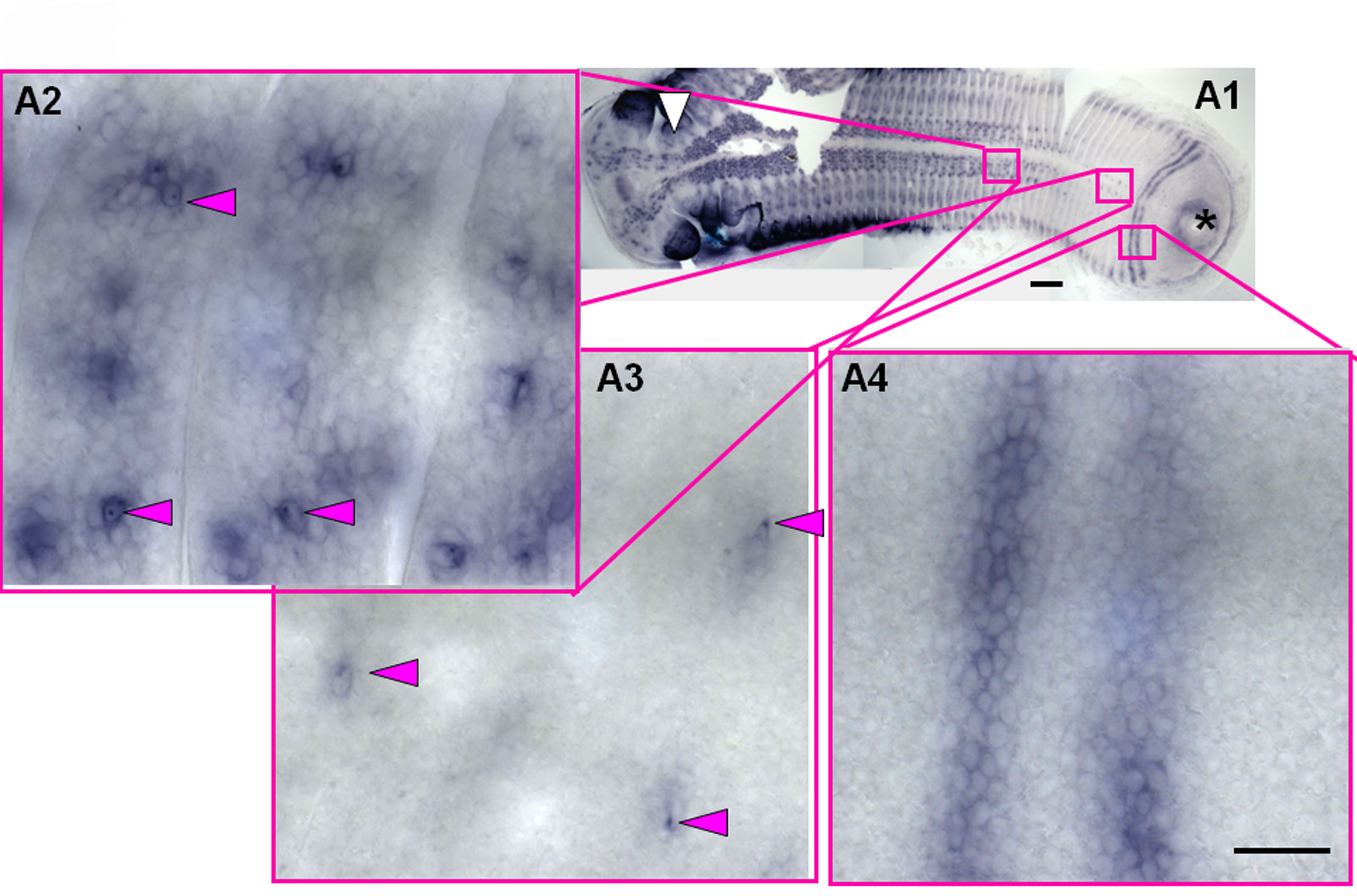

Supplement: Additional file 5: Figure S4 — Distribution of Delta nascent transcripts in an early stage 5 embryo. A1 Flat mount of an embryo at the 41 LBS stage, hybridized with a Delta probe that detects unprocessed nascent transcripts much more strongly than spliced cytoplasmic transcripts (see Additional file 2: Figure S1). At this stage Delta transcripts are no longer detectable in the peri-proctodeal region. Two posterior stripes of Delta expression remain, defined by an accumulation of cytoplasmic transcript, but nuclear dots of nascent transcript are no longer detectable in these stripes (insert A4). Nuclear dots show that transcription of Delta is activated at high level in more anterior medial cells (magenta arrowheads in insert A3). These cells are neuronal precursors. In more mature segments they will form clusters where Delta is present at high level in both the nuclei and the cytoplasm (insert A2). The head is to the left. Black arrowhead: mandibular segment; an asterisk marks the proctodeum. Scale bar in A1: 200 μm, in A2-4: 20 μm. [file 1741-7007-11-112-S5.jpeg]

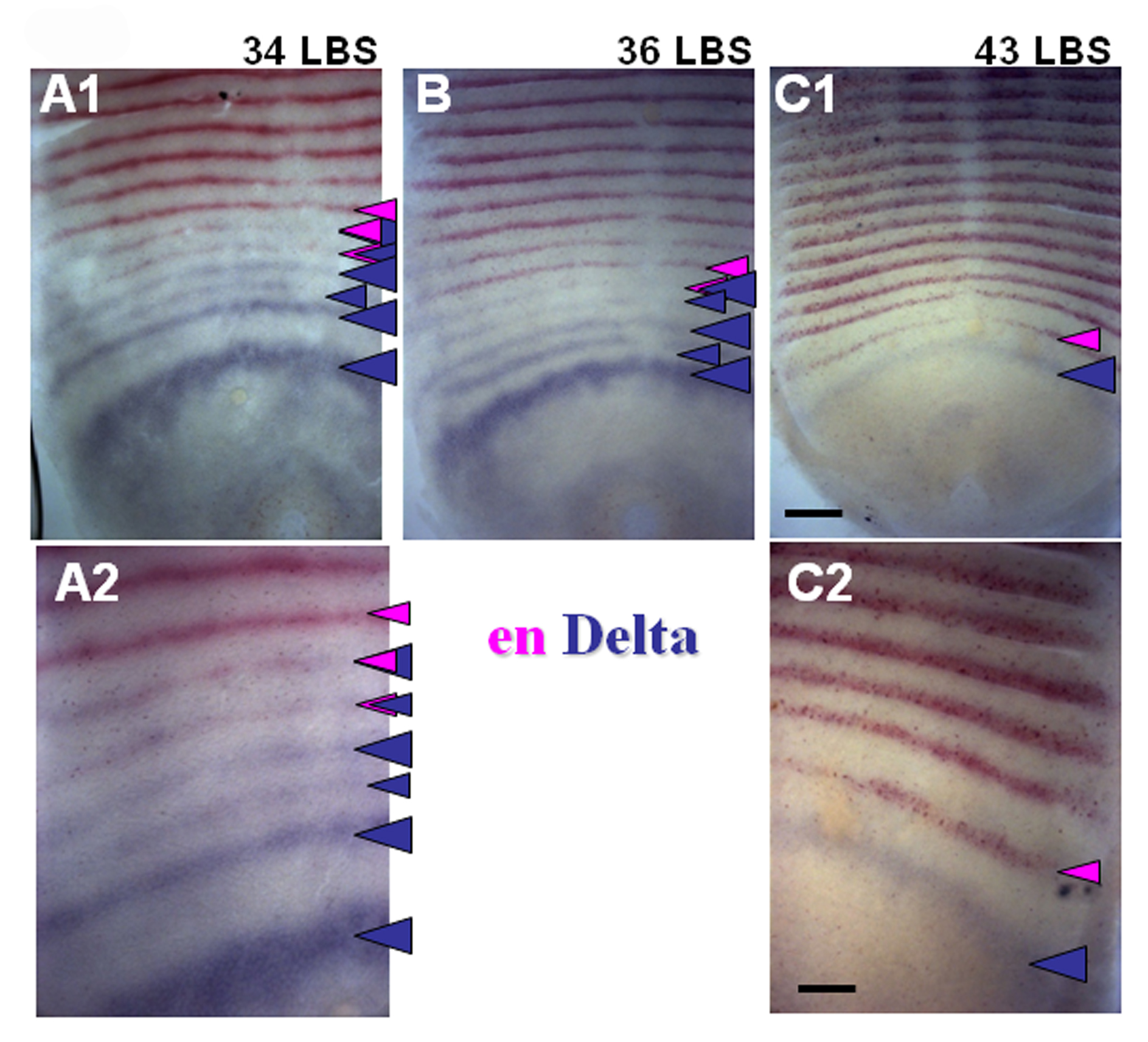

Supplement: Additional file 6: Figure S5 — Last phase of segmentation: from double (primary Delta) to single-segment periodicity (engrailed and secondary Delta). In a 34 LBS embryo (A)Delta still appears at double segment periodicity (large blue arrowheads); intercalated Delta stripes defining the single segment periodicity appear only slightly later, that is, more anteriorly, as the segment pattern matures (small blue arrowheads). engrailed expression (magenta arrowheads) appears yet slightly later than intercalated Delta, overlapping with the segmental stripes of Delta as these fade. engrailed is then expressed persistently in the whole maturing germ band anterior of the segment addition zone. In a 36 LBS embryo (B), the single segment intercalation of Delta appears just after the earliest resolved Delta band; engrailed expression appears closer to this first Delta stripe. By the 43 LBS stage (C), only a single stripe of Delta transcript persists. The transcription of engrailed initiates at a position just one segment anterior to this Delta stripe, but Delta expression has already faded from this anterior region. Scale bar in A1, B1, C1: 100 μm, in A2-C2: 50 μm. [file 1741-7007-11-112-S6.jpeg]

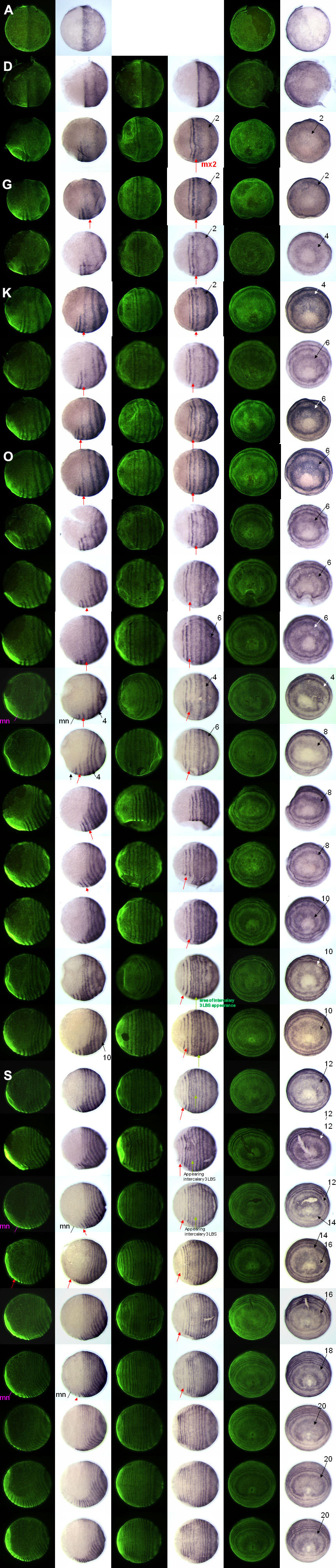

Supplement: Additional file 7: Figure S6 — The complete set of 28 embryos used to deduce the maturing pattern of eve1 expression as presented in main text Figure 10. These embryos have been put into a developmental series using both morphological staging markers and the transitions in the eve pattern itself. Embryos A, D, G, K, O and S are the embryos indicated with the same letters in Figure 10, selected from this full series. The correspondence of bands at the different stages was inferred with reference to the full series, allowing for example the conclusion that the Mx2 band (blue arrow in Figure 10, red arrow in Additional file 7: Figure S6) moves anteriorly during stages 2 and 3. Similar, though less extensive, series were used to infer the correspondence of Delta and eve2 expression patterns between stages. Each embryo is presented in a single row. Columns 1 and 2 - lateral views; columns 2 and 3 - ventral views; columns 4 and 5 posterior views; paired nuclear fluorescence and bright field images. [file 1741-7007-11-112-S7.jpeg]
